# Quantifying H^+^ exchange from muscle cytosolic energy catabolism using metabolite flux and H^+^ coefficients from multiple competitive cation binding: New evidence for consideration in established theories

**DOI:** 10.14814/phy2.14728

**Published:** 2021-04-27

**Authors:** Robert A. Robergs

**Affiliations:** ^1^ School of Exercise and Nutrition Sciences Faculty of Health Queensland University of Technology Kelvin Grove QLD Australia

**Keywords:** acidosis, dissociation constant, glycolysis, intense exercise, lactate, metabolic acidosis, metabolism, pKa, proton exchange

## Abstract

The purpose of this investigation was to present calculations of fractional H^+^ exchange (~H^+^_e_) from the chemical reactions of non‐mitochondrial energy catabolism. Data of muscle pH and metabolite accumulation were based on published research for intense exercise to contractile failure within ~3 min, from which capacities and time profiles were modeled. Data were obtained from prior research for multiple competitive cation dissociation constants of metabolites and the chemical reactions of non‐mitochondrial energy catabolism, and pH dependent calculations of ~H^+^_e_ from specific chemical reactions. Data revealed that the 3 min of intense exercise incurred a total ATP turnover of 142.5 mmol L^−1^, with a total intramuscular ~H^+^ exchange (‐‘ve = release) of −187.9 mmol L^−1^. Total ~H^+^ metabolic consumption was 130.6 mmol L^−1^, revealing a net total ~H^+^_e_ (~H^+^_te_) of −57.3 mmol L^−1^. Lactate production had a ~H^+^_te_ of 44.2 mmol L^−1^ (for a peak accumulation = 45 mmol L^−1^). The net ~H^+^_te_ for the sum of the CK, AK, and AMPD reactions was 36.33 mmol L^−1^. The ~H^+^_te_ from ATP turnover equaled −47.5 mmol L^−1^. The total ~H^+^ release to lactate ratio was 4.3 (187.9/44). Muscle ~H^+^ release during intense exercise is up to ~4‐fold larger than previously assumed based on the lactic acid construct.

## Graphical abstract

Calculations of fractional H^+^ exchange (~H^+^_e_) from the chemical reactions of non‐mitochondrial energy catabolism were performed to quantify the extent of ~H^+^ release during 3 min of intense muscle contractions. The total ~H^+^ release was −187.9 mmol L^−1^ and total ~H^+^ consumption was 130.6 mmol L^−1^. The largest sources of ~H^+^ release were from glycolysis and ATP hydrolysis and the largest contributor to ~H^+^ consumption was lactate production (44.2 mmol L^−1^).

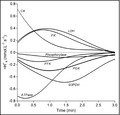

## 1 INTRODUCTION

Historically speaking, the construct of the cellular production of lactic acid, causing a proton (H^+^) release and the development of a metabolic acidosis (lactic acidosis) has been the foundation of our understanding of the development of muscle and systemic metabolic acidosis. For example, the Nobel Prize work of Hill and Meyerhoff in 1922 (Hill, 1926; Hill et al., 1924; Raju, 1998; Shampo & Kyle, 1999) explained the acidosis of intense exercise to be caused by the production of lactic acid as a final by‐product of glycolysis. While no empirical evidence supported or refuted muscle lactic acid production in the 1920s, the ionic form of the acid (lactate) was produced in a variety of organisms and tissues in the absence of oxygen and coincided with the development of acidosis (Holten et al., 1971). Furthermore, and as explained by Robergs et al. (2004) but overlooked by Ferguson et al. (2018), a historical account of scientific inquiry into muscle metabolism at this time was based on an understanding of metabolism without the knowledge of the computational chemistry of H^+^ association and dissociation. As such, from the perspectives of Hill, Meyerhoff and indeed all scientists of that era and for many decades later, lactate and lactic acid were synonymous.

We now know, thanks to the work of Henderson in the 1940s and the extensive research of the more detailed quantification of pH dependent molecular H^+^ dissociation that followed (NIST, 1994), that lactate production is a metabolic fractional (~) H^+^ (~H^+^) consumer and as such opposes not contributes to metabolic ~H^+^ release. For example, Robergs et al. (2004) presented explanations of the organic chemistry of the reactions of skeletal muscle non‐mitochondrial energy catabolism and demonstrated that all ‘acids’ produced from these reactions are produced in their ionized state. In other words, there is never the production of a strong acid (such as pyruvic acid or lactic acid) in cellular energy catabolism. As such, it is biochemically impossible for the cellular production of lactic acid, and the added reality is that the organic chemistry of the lactate dehydrogenase reaction reveals that lactate production consumes not releases a ~H^+^ (Robergs et al., 2004).

There is a long history of scientific opposition to the lactic acidosis construct (Dennis et al., 1991; Gevers, 1977; Gevers, 1979; Hochacka & Mommsen, 1983; Robergs, 2001; Robergs et al., 2004; Robergs et al., 2005; Robergs et al., 2006; Robergs, 2017; Robergs, 2019; Zilva, 1978). As early as 1977 Gevers (1977) questioned the construct of a lactic acidosis and presented the biochemical evidence for disproving cellular lactic acid production. At this time Gevers proposed that ATP hydrolysis in excess of ATP regeneration from oxidative phosphorylation increased net H^+^ release and was the likely source of the H^+^ that contributed to metabolic acidosis. Such a biochemical understanding of cellular metabolism during metabolic demand that causes increased glycolysis and the need for lactate production to sustain cytosolic NAD^+^ regeneration was further supported by Zilva (1978), Dennis et al. (1991) and Hochachka and Mommsen (1983). The latter authors clearly stated and documented the rationale for pH dependent ~H^+^_e_ in chemical reactions and the need for biochemistry textbooks to stop presenting whole numbers for H^+^ involvement in chemical reactions (Hochachka & Mommsen, 1983).

Despite the clarity of the empirical evidence disproving cellular lactic acid production, the commentary by Robergs et al. (2004) received criticism based on an over‐simplistic presentation and accounting of H^+^ balance for the reactions included in their analyses (Boning et al., 2005; Kemp, 2005; Kemp et al., 2006). Although this issue was secondary to and of no relevance to the issue of disproving a lactic acidosis, such criticism was warranted as the ~H^+^_e_ during chemical reactions is influenced by cellular pH, as well as the presence of competing cations to H^+^ (Mg^+2^, Na^+^, K^+^, Ca^+2^) for binding with ionizable groups of metabolites (Kushmerick, 1997; Robergs, 2019; Robergs et al., 2004; Vinnakota et al., 2006). Furthermore, it is important to know that the ~H^+^_e_ during chemical reactions that involve a H^+^ as a substrate or product is fractional (hardly ever +1 or −1) and many reactions presented in biochemical textbooks do not present ~H^+^ balanced chemical reactions, thereby misleading readers of the actual involvement of ~H^+^ release or consumption in many reactions of cytosolic energy catabolism. Even reactions that do not directly involve covalent H^+^_e_ still have ~H^+^_e_ due to differences in weak ~H^+^ dissociation or association between the substrates and/or products of the reaction across the physiological pH range (Robergs, 2019).

In response to the prior criticisms, Robergs (2017) quantified the competitive cation adjusted ~H^+^_e_ for all reactions of muscle non‐mitochondrial energy catabolism. Such data provided accurate computations of the pH dependent ~H^+^ release or metabolic consumption for the 17 reactions of non‐mitochondrial energy catabolism, which in turn involved 21 metabolites, and 104 metabolite‐cation complexes. Such research revealed the extent of net ~H^+^ release from glycolysis, that the glyceraldehyde‐3‐phosphate dehydrogenase reaction was the most ~H^+^ releasing reaction of non‐mitochondrial energy catabolism and increases in ~H^+^ release as pH decreases, and that ATP hydrolysis is the second most ~H^+^ releasing reaction, although this propensity decreases as pH decreases. Of equal importance, the lactate dehydrogenase reaction is always ~H^+^ consuming and this propensity is relatively pH independent across the physiological pH range, varying from a ~H^+^_e_ of 0.9995 to 0.9954 from pH 7.0 to 6.0 respectively (Robergs, 2017).

The refutation of the lactic acidosis construct has left the physico‐chemical theory of acidosis as the remaining paradigm that might explain acid‐base disturbances in biological systems. Central to the main method of the physico‐chemical theory of acidosis is the concept of the strong ion difference (SID) as proposed by Stewart (Stewart, 1978, 1983). This approach assumes that the principles of electrochemical neutrality govern the change in charged compounds and elements in a biological solution. As such, the pH of a biological solution is totally dependent on disturbances in charge, which in turn means that the [H^+^] of a biological solution is a totally dependent variable. Such a theory means that there is no direct H^+^ contributions to cellular or systemic H^+^ balance. For example, as stated by Stewart, “*… H^+^ movements into or out of a solution do not provide quantitative explanations for changes in [H^+^]*.” (Stewart, 1983, p. 1445). Yet Stewart never provided any external data of acid‐base balance in biological solutions to validate his theory. Clearly, computation of the ~H^+^_e_ from cellular catabolism during times of increased ATP demand is essential to critically challenge or support the current Stewart approach to understanding the physico‐chemical influences to perturbations of the acid‐base balance of biological solutions.

Regardless of the mechanism(s) accounting for cellular metabolic acidosis, the last 80 years of research inquiry of muscle metabolic acidosis during intense exercise documents a clear cellular acidosis that directly alters systemic (blood and interstitial fluid) acidosis by multiple transport mechanisms of H^+^ from muscle to blood (Juel et al., 1990, 2004). The magnitude and temporal profile of the decrease in muscle pH during intense exercise is important to the computation of pH dependent ~H^+^_e_ as explained in Methods. As such, it is important to note the research that has informed us of the influence of cellular catabolism during intense exercise to cellular pH. Such research is based largely on methodologies of muscle biopsy and more recently on 31‐phosphorous magnetic resonance spectroscopy (^31^P MRS), though each method has its own limitations as explained in the Discussion.

Muscle pH at rest approximates 7.0 (Taylor et al., 1986). At the onset of intense exercise there is a slight increase in muscle pH, which has been attributed to the ~H^+^_e_ of the creatine kinase (CK) reaction prior to the meaningful activation of and substrate flux through the glycolytic pathway (Cannon et al., 2014; Haseler et al., 1998; McCann et al., 1995; Moll et al., 2017; Slade et al., 2006; Yoshida & Watari, 1992). Thereafter, the growing anaerobic ATP turnover fueled further by the CK reaction and now a rapidly increasing substrate flux through glycolysis combine to increase net ~H^+^ release, which also coincides with an increasing intramuscular Pi accumulation (Bangsbo et al., 1993; Hagberg, 1985; Kemp et al., 2007; Taylor et al., 1986; Wackerhage et al., 1996; Wilson et al., 1988), increased K^+^, lactate and H^+^ efflux (Juel et al., 1990, 2004), and a decrease in cellular and blood pH (Cannon et al., 2014; Juel et al., 2004; Yoshida & Watari, 1992). Different forms of intense exercise and exercise durations yield different end‐exercise muscle pH values, but such extremes of cellular acidosis have been documented as low as 6.5 from muscle biopsy for repeated bouts of maximal isokinetic cycling (Spriet et al., 1989), 6.28 from muscle biopsy of repeated intense human muscle contraction induced by artificial electrical stimulation during anoxia (Spriet et al., 1987b), and 5.86 from ^31^P MRS during severe intensity forearm wrist flexion exercise (Wilson et al., 1988).

Given the continued need to more accurately assess H^+^ balance in skeletal muscle during intense exercise, the purpose of this research was to quantify the ~H^+^_e_ during non‐mitochondrial energy catabolism in contracting skeletal muscle during intense exercise. The data were used for a presentation at the American Physiological Society Intersociety Meeting in New Orleans, October, 2018. Based on the presentation, the author was invited to write and submit a review manuscript that included some of the modelled data. This work was published in a very simplified and constrained presentation based on an altered exercise duration and consequently a different data set (Robergs, 2019), leaving this manuscript as the source of the more detailed research methodology, data, results, and discussion. To accomplish this, data from prior research was used to obtain the ~H^+^ balance of all reactions of non‐mitochondrial energy metabolism, adjusted for competitive cation binding (Robergs, 2017). These data were combined with data of metabolite accumulation along with temporal profiles (modelling) of pertinent metabolites to allow the quantification of ~H^+^ release and consumption during 3 min of intense anoxic exercise. Such computations of ~H^+^_e_ in contracting skeletal muscle for a specific intense exercise condition should provide the most accurate account of the capacities for direct H^+^ involvement in cellular and systemic acid‐base balance during non‐mitochondrial energy metabolism presented to date, quantify the ~H^+^ release from specific reactions of glycolysis and reveal the quantity and reaction source of the main contributors to ~H^+^ release during intense exercise. Such an account of total ~H^+^_e_ would then provide an accurate quantity for the extent of the non‐stoichiometric relationship between total ~H^+^ release and lactate production.

## 2 MATERIALS AND METHODS

The list of metabolites and reactions included in this computational evaluation of skeletal muscle ~H^+^_e_ from non‐mitochondrial energy catabolism during intense exercise were taken from Robergs et al. (2004) and are presented in Table 1. To aid understanding of substrate flux through these reactions, data from prior research was obtained to profile the change in each metabolite during 3 min of intense exercise to fatigue in anoxia (either induced experimentally or assumed based on the high intramuscular pressures generated during the intense exercise). The closest prior research to these conditions was that of Spriet et al. (1987a, 1987b) which involved electrical stimulation to the quadriceps musculature (rectus femoris) with femoral artery occlusion for a total duration of 3.4 min based on repeated intervals of 1.6 s of square wave pulses of 0.5 ms at 20 Hz, separated by rest intervals of 1.6 s (time during contractions = 102.4 s). This research also provided data for muscle ATP turnover (ATP_to_), which was further compared to the data for ATP_to_ from Bangsbo et al. (1990) and Medbo and Tabata (1993). The ATP_to_ data from this prior research (rate and integrated total) were used as an external validation of the computed data for ATP_to_ from this research.

**TABLE 1:** The chemical reactions of muscle phosphagen and glycolytic energy systems

Reaction	Enzyme
ATP hydrolysis	
ATP + H_2_O ↔ ADP + Pi + ~H^+^	ATPase
Phosphagen system	
HCrP + ADP + ~H^+^ ↔ Cr + ATP	Creatine kinase (CK)
ADP + ADP ↔ ATP + AMP	Adenylate kinase (AK)
AMP + ~H^+^ ↔ IMP + NH_4_	AMP deaminase (AMPD)
Glycogenolysis	
Glycogen(n) + HP ↔ Glycogen (n − 1) + G1P	Phosphorylase
G1P ↔ G6P	Phosphoglucomutase (PGluM)
Glycolysis	
Glucose + ATP ↔ G6P + ADP + ~H^+^	Hexokinase^*^
G6P ↔ F6P	Glucose‐6‐phosphate isomerase (G6PI)
F6P + ATP ↔ F1,6P + ADP + ~H^+^	Phosphofructokinase (PFK)
F1,6P ↔ DHP + G3P	Aldolase (Ald)
DHP ↔ G3P	Triosephosphate Isomerase (TPI)
G3P + HPi + NAD^+^ ↔ 1,3BPG + NADH + ~H^+^	Glyceraldehyde‐3‐phosphate dehydrogenase (G3PDH)
1,3BPG + ADP ↔ 3PG + ATP	Phosphoglycerate kinase (PGK)
3PG ↔ 2PG	Phosphoglycerate mutase (PGlyM)
2PG ↔ PEP + H_2_O	Enolase (Enol)
PEP + ADP + ~H^+^ ↔ Pyr + ATP	Pyruvate kinase (PK)
Lactate production	
Pyr + NADH + ~H^+^ ↔ La + NAD^+^	Lactate dehydrogenase (LDH)

Note

~ represents fractional; *This reaction was not included in the calculations due to the ischemic hypoxia conditions of the model. Note that reactions are not presented balanced by charge or ~H^+^ as these are not constants or whole numbers due to their pH dependence. Also note that reactions with no covalent H^+^ component can still have ~H^+^ exchange due to the specific features of the substrate and product H^+^ association/dissociation. NAD^+^ = nicotinamide adenine dinucleotide (oxidized form); NADH = nicotinamide adenine dinucleotide (reduced form); See Table 2 for definitions of all other metabolite abbreviations.

Data for changing muscle ATP, CrP, Cr, Pi, lactate, ADP, AMP, ammonia, glycolytic intermediates, and pH were obtained from multiple prior research studies (Bangsbo, 1998; Bangsbo et al., 1990; Banagsbo et al., 1993; Cannon et al., 2014; Gray et al., 2011; Jones et al., 1985; Kemp, 1997, 2001; Kemp et al., 2006, 2007; McCartney et al., 1986; Medbo & Tabata, 1993; Sahlin, 1978, 1985; Sahlin et al., 1976, 1981, 1987, 1998; Sahlin & Henriksson, 1984; Spriet, 1992; Spriet et al., 1985, 1987a, 1989; Wackerhage et al., 1996; Yoshida & Watari, 1992). Metabolite concentrations were converted from either of mmol kg^−1^ dry wt. or mmol kg^−1^ wet wt. to mmol L^−1^ muscle water based on the assumption of the cellular mass being 76% water (Equations 1 and 2) (Kemp et al., 2007).

(1) $$\left[\text{metabolite}\right]\text{mmol}\;{\text{kg}}^{‐1}\;\text{wet wt}.=\left[\text{metabolite}\right]\text{mmol}\;{\text{kg}}^{‐1}\;\text{dry wt}.\times \left(1‐0.76\right),$$

(2) $$\left[\text{metabolite}\right]\text{mmol}\;L^{‐1}\;\text{muscle water}=\left[\text{metabolite}\right]\text{mmol}\;{\text{kg}}^{‐1}\;\text{wet wt}./0.76\text{.}$$

**FIGURE 1:**
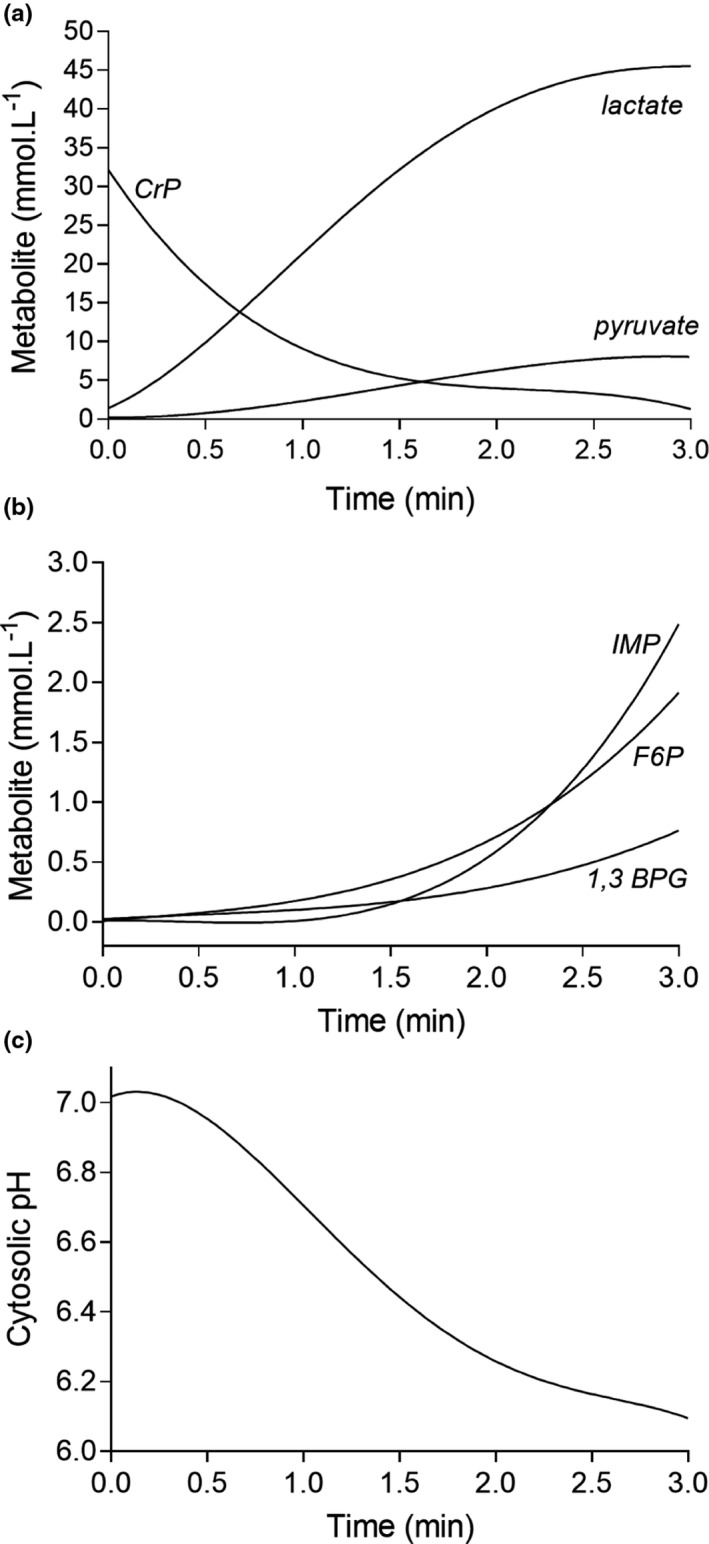
Modeled changes in some of the main muscle metabolites of interest. (a) lactate, pyruvate, and creatine phosphate (CrP); (b) inosine monophosphate (IMP), fructose‐6‐phosphate (F6P), and 1,3 bisphosphoglycerate (1,3 BPG); (c) cytosolic pH. See Table 2 for the polynomial functions. See Methods for the importance of the lactate curve in computing substrate flux through glycolysis

While the assumed metabolite accumulation and therefore production curves for this research are all important, the two most important components of the model are the changes in muscle pH and the lactate concentration. As explained in the Introduction, the profile of the change in muscle pH during sustained intense exercise was based on prior research, with clear evidence for the physiological range in muscle pH being from 7.0 to 6.0 for pertinent exercise conditions (Spriet et al., 1987b, 1989; Wilson et al., 1988; Yoshida & Watari, 1992) (Figure 1c). As will be explained, this pH data were used to compute the pH dependent H^+^ association and dissociation for all metabolites of Table 1, which in turn were impactful to the final calculations of ~H^+^_e_.

The capacity of muscle lactate production was assumed to equal the lactate accumulation in muscle during ischemic anoxia. To ascertain the instantaneous change in muscle lactate, as indeed for all metabolites of non‐mitochondrial energy catabolism and also pH, non‐linear functions were developed for each metabolite to profile the change from rest to concentrations spanning the 3 min duration (Figure 1). To accomplish this, metabolite data were graphed and fitted to pertinent 3^rd^ or 4^th^ order polynomial functions to derive equations (Prism, GraphPad Software) (Table 2). For lactate, a sigmoidal model was applied where lactate first increased as a slow exponential function from a rest value of 1.25 mmol L^−1^, and then in a near linear rapid function from 0.5 to 1.5 min (Figure 1a). Thereafter, lactate production (slope) gradually decreased as muscle contractile failure and reduced ATP turnover progressed. The peak increase in muscle lactate (flux time integral) was assumed to be 44 mmol L^−1^ based on the results from Spriet et al. (1987a).

**TABLE 2:** Polynomial equations of the temporal profiles of metabolite accumulation for the exercise condition

Metabolite	Polynomial equation coefficients* A,B,C,D,E
Acidosis	
↓pH	7.017, 0.2107, −0.8556, 0.385, −0.05263
Phosphagen system	
↓CrP; creatine phosphate	32.15, −37.19, 16.73, −2.587, 0
↑Pi; inorganic phosphate	7.218, −0.6074, 2.347, 1.515, −0.5128
↑NH_3_; ammonia	0.07147, −0.133, 0.4369, 0.01492, 0
↑ADP; adenosine diphosphate	0.005596, −0.004046, 0.006386, −1.865 e−005, 0
↑AMP; adenosine monophosphate	0.0004856, 0.0005484, −0.0001652, 0.0001594, 0
↑IMP; inosine monophosphate	0.011, 0.03363, −0.1854, 0.1497, 0
↓ATP; adenosine triphosphate	9.991, 0.002628, 0.1442, −0.1651, 0
Glycogenolysis	
↓Glycogen	200, −4.657, −74.62, 38.03, −5.443
↑G1P; glucose‐1‐phosphate	0.009162, 0.1559, −0.1208, 0.074131, 0
↑Glucose^	0.009162, 0.1559, −0.1208, 0.074131, 0
↑G6P; glucose‐6‐phosphate	0.5048, −0.007611, −0.01245, 0.02387, 0
Glycolysis	
↑F6P; fructose‐6‐phosphate	0.007912, 0.141, −0.04324, 0.06937, 0
↑F16P; fructose‐1,6‐bisphosphate	0.009162, 0.1559, −0.1208, 0.074131, 0
↑DHP; dihydroxyacetone phosphate	0.009162, 0.1559, −0.1208, 0.074131, 0
↑G3P; glyceraldehyde‐3‐phosphate	0.009162, 0.1559, −0.1208, 0.074131, 0
↑1,3BPG; 1,3‐bisphosphoglycerate	0.02416, 0.08844, −0.0433, 0.03198, 0
↑3PG; 3‐phosphoglycerate	0.009162, 0.1559, −0.1208, 0.074131, 0
↑2PG; 2‐phosphoglycerate	0.009162, 0.1559, −0.1208, 0.074131, 0
↑PEP; phosphoenolpyruvate	0.009162, 0.1559, −0.1208, 0.074131, 0
↑Pyr; pyruvate	0.238, 0.7047, −1.129, 0.5893, 0
Lactate production	
↑La; lactate	1.424, 10.77, 15.87, −7.654, 0.9333

Note

*x = time (min); ^The hexokinase reaction was not included in calculations; [metabolite] = A + Bx + Cx^2^ + Dx^3^ + Ex^4^.

All remaining computations were performed from custom software (LabVIEW™, National Instruments). The polynomial coefficients for the accumulation of each metabolite and also the pH dependent fractional H^+^ exchange coefficients (~H^+^) of each reaction from Robergs et al. (2017) were imported into the software. Metabolite data were then corrected based on the subtraction of resting values (ΔM) (Equation 3) and then calculation of the increment concentrations (M_incr_) at 1 Hz (Equation 4).

To compute metabolite accumulation (M_a_), which was assumed to represent production in this anoxic model, the increment metabolite data were adjusted based on the equations of Table 3, where for single independent reactions, the M_a_ was equal to the accumulation (or breakdown) of pertinent substrates or products based on the integral of the increment data (Equation 5). For reactions that were singular and therefore not a part of glycogenolysis or glycolysis, M_a_ was equivalent to metabolic flux (M_f_). Noted examples of this approach are for the LDH and CK reactions. For reactions involved in sequential multiple reaction pathways, M_f_ was based on adjustment to the final accumulating product of the pathway. For glycolysis during the modeled ATP demand of this study, this was lactate. As such, the muscle lactate accumulation data were used to derive all other metabolite flux calculations from glycogenolysis and glycolysis, as presented in Table 3. For example, for the reaction preceding lactate production, that of pyruvate kinase, M_f_ was quantified by adding the profiled M_a_ of muscle pyruvate accumulation to the M_f_ of the LDH reaction (for LDH, M_a_ = M_f_). This pyruvate kinase M_f_ was then added to the M_a_ of phosphoenolpyruvate to compute the enolase reaction M_f_. The enolase M_f_ was then used as the core component of the preceding reaction, as shown in the equations of Table 3. This approach was replicated sequentially in reverse along the glycolytic and glycogenolytic pathways at a computational interval of 1 Hz (Table 3, Equations i–xxi).

**TABLE 3:** Computation formulae for the metabolite capacity (flux; M_f_) of the reactions of the muscle cytosolic phosphagen and glycolytic energy systems^a^, with data results for fractional H^+^ coefficients (~H^+^) and total fractional exchange (~H^+^_te_)

Reaction	Equation (flux; mmol L^−1^)	Eq'n	Reaction ~H^+^		M_tf_ ^b^	~H^+^_te_
			*pH = 7*	*pH = 6*		
Phosphagen system						
Creatine kinase	CK_f_ = Δ[CK]	i	0.9690	0.0823	31.32	29.65
Adenylate kinase	AK_f_ = Δ[AMP] + Δ[IMP]	ii	−0.0209	−0.1281	6.31	−0.40
AMP deaminase	AMPD_f_ = Δ[IMP]	iii	1.0877	1.2029	6.21	7.08
Glycogenolysis						
Phosphorylase	Phosphorylase_f_ = PGluM_f_ + Δ[G1P]	iv	−0.2029	−0.2922	34.98	−11.00
Phosphoglucomutase	PGluM_f_ = G6PI_f_ + Δ[G6P]	v	0.0168	0.0441	33.59	0.90
*Glycogenolysis total*			−0.1861	−0.2481		−10.1
Glycolysis						
Hexokinase	Not included					
Glucose‐6‐phosphate isomerase	G6PI_f_ = PFK_f_ + Δ[F6P]	vi	−0.0421	−0.1260	33.08	−4.64
Phosphofructokinase	PFK_f_ = (Ald_f_) + Δ[F1,6BP]	vii	−0.7350	−0.0160	31.18	−13.81
Aldolase	Ald_f_ = TPI_f_ = (G3PD_f_/2) + ((Δ[G3P] + Δ[DHP])/2)	viii	−0.0087	−0.0664	29.80	−0.95
Triosephosphate Isomerase		ix	0.1478	0.3009	29.80	8.06
Glyceraldehyde‐3‐phosphate dehydrogenase	G3PDH_f_ = PGK_f_ + Δ[1,3BPG]	x	−0.7603	−1.5798	56.83	−61.51
Phosphoglycerate kinase	PGK_f_ = PGlyM_f_ + Δ[3PG]	xi	−0.6565	−0.5230	56.09	−34.68
Phosphoglycerate mutase	PGlyM_f_ = Enol_f_ + Δ[2PG]	xii	0.1114	0.1515	54.70	2.76
Enolase	Enol_f_ = PK_f_ + Δ[PEP]	xiii	−0.1702	−0.3008	53.23	−13.41
Pyruvate kinase	PK_f_ = LDH_f_ + Δ[Pyruvate]	xiv	0.8830	0.3516	51.94	37.90
*Glycolysis total*						−80.28
Lactate production						
Lactate dehydrogenase	LDH_f_ = Δ[Lac]	xv	1.0004	1.0044	44.08	44.23
ATPase; for total ATP_to_	ATPase_f_ = Δ[ATP] + (∑eq'n i,ii,xi,xiv)	xvi			142.46	
for ~H^+^_e_	ATPase_f_ = Δ[ATP] + (∑eq'n i,ii,xi,xiv) − eq'n vii		−0.6635	−0.0097	111.35	−56.39
Glycogenolysis, glycolysis & lactate production						−46.15
delta NAD^+^		xvii			12.66	
delta ADP		xviii			24.73	
net Pi		xix			19.74	
total H^+^ release		xx				−187.86
H^+^ release/La ratio		xxi				4.3

Note

Δ = absolute number for the change in metabolite concentration from rest.

^a^ Note that the hexokinase reaction was not included in calculations due to the anoxic nature of the exercise model. The M_tf_ and ~H^+^_te_ data are results as explained in the Methods and Results sections.

^b^ M_tf_ data only provided for single reactions (mmol L^−1^). ~H^+^_e_ data (mmol L^−1^).

Total metabolite flux (M_tf_) was the integral of the M_f_ data corrected for carbon numbers, the multiple reactions of aldolase and TPI at the start of phase‐II of glycolysis, as well as for the summation of the AK and AMPD reactions to account for flux through the AK reaction. Note that for several reactions, where limited data exist for their accumulation in skeletal muscle, metabolite accumulation for G1P, F1,6P, DHP, G3P, 3PG, 2PG, and PEP were assumed to fit the same profile.

(3) $$\text{Metabolite accumulation}\;\left(\Delta M\right)=\left({\left[M\right]}_{i}‐{\left[M\right]}_{\text{rest}}\right),$$

(4) $$\text{Metabolite increment}\left(M_{\text{incr}}\right)=\left(\left(\Delta M_{n+1}\right)‐\left(\Delta M_{n}\right)\right).$$

(5) $$\text{Total metabolite flux}\left(M_{\text{tf}}\right)=\int_{i}^{n}M_{f}\;\text{dt}.$$

The calculation of M_tf_ through the 3 min exercise condition allowed for the increment (1 Hz) pH dependent calculation of the ~H^+^ exchange (H^+^_e_). For this, the M_f_ data were multiplied by the pH dependent ~H^+^ coefficient data (42) for the given pH at each data point, and then each ~H^+^_e_ metabolite array for the 3 min exercise condition was integrated to yield the total ~H^+^_e_ (H^+^_te_) for the specific reaction (Equation 6). The only exception to this process was for the ~H^+^_e_ of ATP hydrolysis in the family of cellular ATPase reactions. To compute an accurate ~H^+^_e_ from ATP hydrolysis, the total ATP_to_ flux data had to be adjusted for the ATP cost of the PFK reaction, as the ~H^+^_e_ for this subset of ATP_to_ is accounted for in the calculations from the PFK reaction. All data sets for ΔM, M_incr_, M_f_, M_tf_, ~H^+^_e_, and ~H^+^_te_ were saved and imported into a graphics program for curve fitting and presentation in figure format (GraphPad Prism version 7.0 for Windows, GraphPad Software).

(6) $$\text{Reaction total}\left(\text{integral}\right){\text{fractional H}}^{+}\;\text{exchange}\left(∼H_{\text{te}}^{+}\right)=\int_{i}^{n}(M_{f}\cdot ∼H^{+})\;\text{dt}.$$

For muscle ATP_to_, the flux of substrate through the reactions that regenerate ATP were summed to produce total ATP_to_ (Table 3). A similar strategy was used for the production of NADH and NAD^+^ (NAD^+^ balance; Equation 7) to assess relative changes in cytosolic redox (Equation 8), as well as for ADP (Equation 9) to assess the capacity of ADP production and ascertain the disposal of ADP needed to keep muscle [ADP] to its low values as verified by research from muscle biopsy and calculations from ^31^P MRS.

(7) $${\text{NAD}}_{\text{balance}}^{+}=\int_{i}^{n}\left(\left({\text{LDH}}_{f}\right)‐\left({\text{G3PDH}}_{f}\right)\right)\;\text{dt},$$

(8) $$\text{cytosolic redox}=\left[{\text{NAD}}^{+}\right]/\left[\text{NADH}\right],$$

(9) $${\text{ADP}}_{\text{balance}}=\int_{i}^{n}\left(\left({\text{ATP}}_{\text{to}}\right)+\left({\text{PFK}}_{f}\right)‐\left({\text{PGK}}_{f}\right)‐\left({\text{PK}}_{f}\right)‐\left({\text{CK}}_{f}\right)‐\left(2\;{\text{AK}}_{f}\right)\right)\;\text{dt},$$

(10) $$\text{Buffer capacity}\left(\text{Slykes}\right)=\text{total}∼H^{+}\;\text{release}/\Delta \text{pH}.$$

The H^+^ buffer capacity comprises metabolic buffering from the ~H^+^ association of metabolites. Based on the work of Robergs (2017), we can now compute these ~H^+^ values for specific reactions and metabolites at a known pH. However, we do not know the full extent of such buffering as we do not know the capacity of H^+^ association for the remainder of the negatively charged polar molecules within a cell. As such, H^+^ buffering capacity is estimated from knowing the capacity for H^+^ release compared to the measured change in system (e.g. for a specific tissue such as muscle, or blood) pH. This mathematical relationship is expressed in Equation (10), and the unit used to quantify this entity is the Slyke in honor of Donald Van Slyke (1883–1971), a Dutch‐American biochemist who pioneered extensive research of amino acid structures and the computational chemistry of gases in solution and acid (H^+^) buffering (Hastings & Van Slyke, 1976).

## 3 RESULTS

The muscle M_a_ curves for the main chemical reactions of interest are presented in Figure 1a–c, and all polynomial functions for the accumulation of all metabolites were presented in Table 2. Once again, from a methodological viewpoint for this modeled exercise condition, the most important M_a_ curve was for muscle lactate ([La^−^]) as this (after baseline correction) equaled the LDH M_f_ and was the basis for M_f_ and M_tf_ estimation of all reactions of glycolysis (Table 3). The CK reaction was additionally impactful as the baseline corrected CK M_a_ also equaled M_f_. The modelled metabolites and pH profile of the exercise condition allowed for high temporal resolution calculation of pH dependent ~H^+^_e_ for all data points (1 Hz for 3 min) and chemical reactions, and the subsequent H^+^_e_ and H^+^_te_ (Equation 6) for all reactions.

**FIGURE 2:**
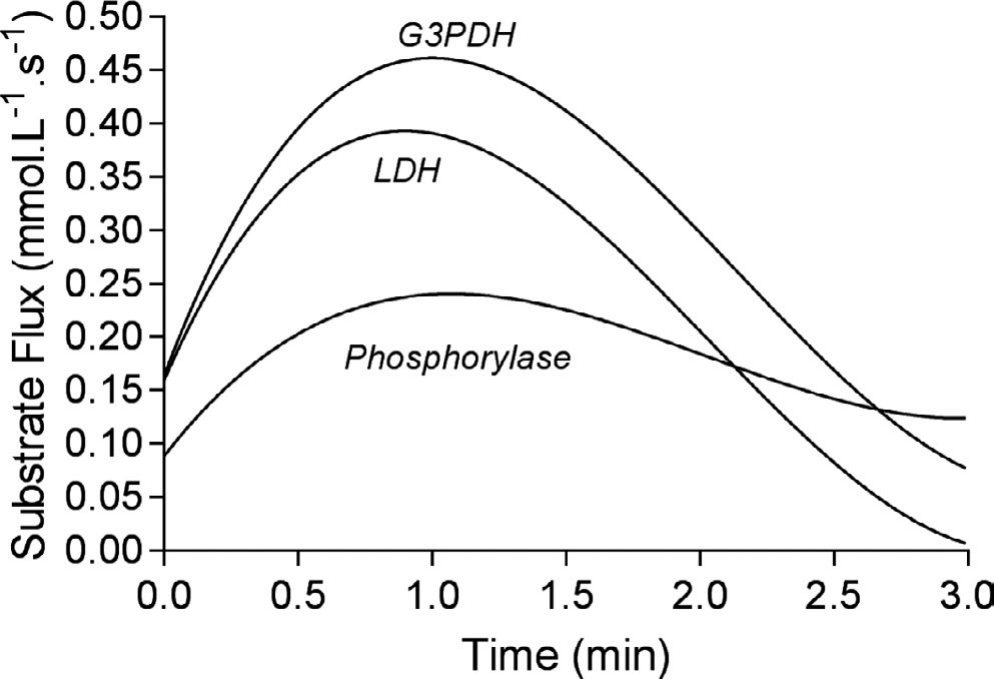
Calculated metabolite flux (M_f_) for lactate (La^−^) and products of select reactions spanning glycogenolysis (phosphorylase reaction), phase‐1 of glycolysis (G3PDH = glyceraldehyde‐3‐phosphate dehydrogenase reaction) and phase‐II of glycolysis (LDH = lactate dehydrogenase reaction)

**FIGURE 3:**
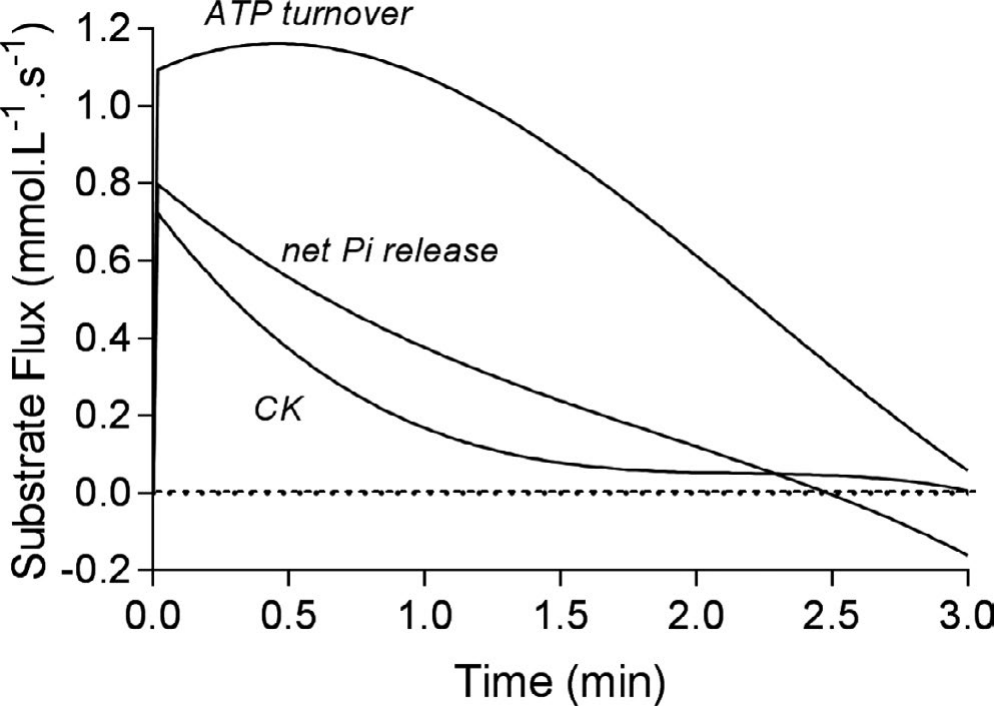
Changes in calculated metabolite flux (M_f_) for (a) muscle non‐mitochondrial ATP turnover, (b) creatine phosphate (CrP) breakdown (CK reaction), and (c) net (release − consumption) Pi release during the exercise condition

The M_f_ curves for pertinent metabolites are presented in Figures 2 and 3, where the *y*‐axis represents the rate of product formation. ATP turnover (ATP_to_) was able to be calculated from the M_f_ of the ATP regenerating reactions (CK, AK, PGK, and PK) added to the temporal profile of the decrease in muscle [ATP] (Figure 3). The difference between the CK_f_ and ATP_to_ curves of Figure 3 predominantly represents the ATP contribution from glycolysis. Peak ATP_to_ equaled 1.161 mmol L^−1^ s^−1^, total ATP_to_ (integral of the ATP_to_ curve) equated to 142.46 mmol L^−1^, and PFK reaction corrected ATP_to_ equated to 111.35 mmol L^−1^. The net release of inorganic phosphate (Pi) (19.74 mmol L^−1^) is the balance of Pi release from the PFK reaction corrected ATP_to_ minus the Pi consumption in the phosphorylase and G3PDH reactions which equaled 91.61 mmol L^−1^. This simultaneous Pi release and metabolic consumption during cytosolic energy catabolism adds complexity to understanding the role of Pi as a H^+^ buffer, as will be explained in the Discussion. Also, note that the negative data for Pi release in Figure 3 reveal that after 2.5 min, more Pi was metabolically consumed then released even though the prior release causes an appreciable net Pi M_a_.

**FIGURE 4:**
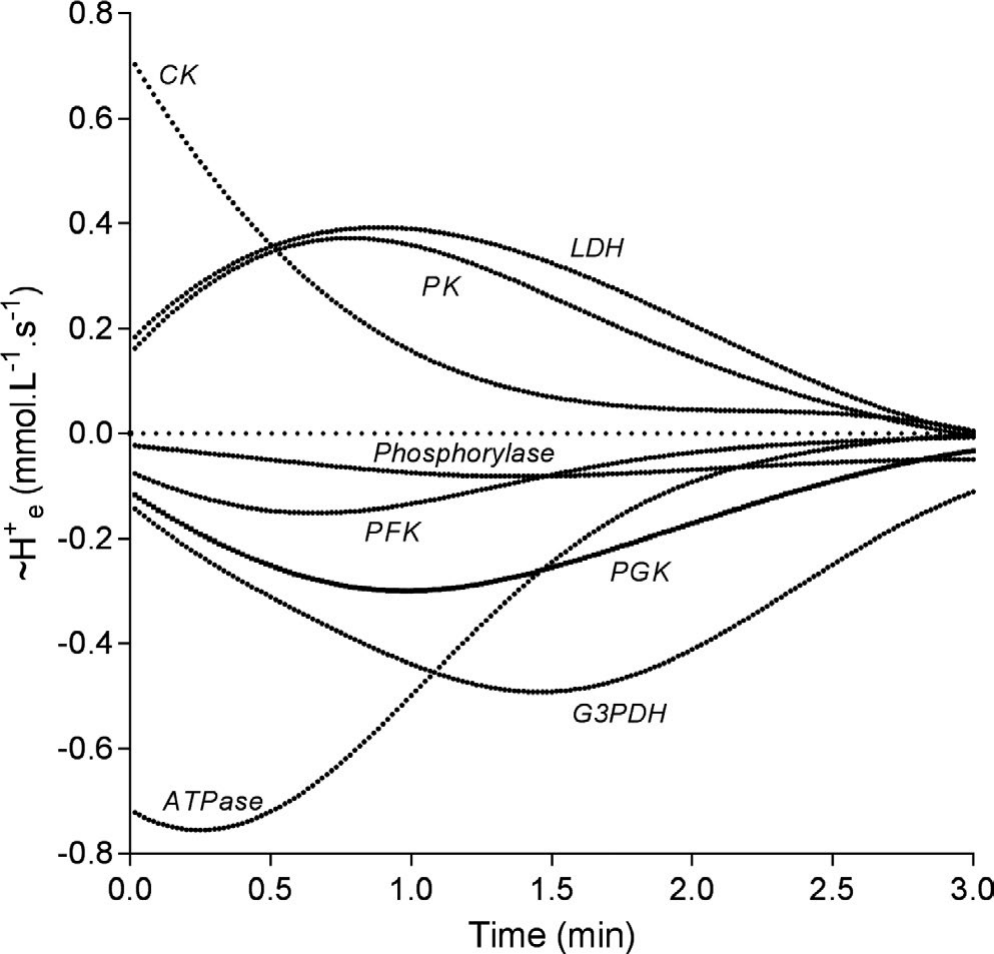
The temporal profile of pH dependent multiple cation competitive fractional H^+^ exchange (~H^+^_e_) for the pertinent reactions of non‐mitochondrial energy catabolism. Negative data equals ~H^+^ release

The final curves based on M_f_ and H^+^_e_ did not conform to a standard polynomial function of any order. Consequently, figure data of the temporal profiles of the pH dependent H^+^_e_ for the main reactions of interest (Figure 4) are presented as data points at a frequency of 1 Hz. Note the sustained ~H^+^_e_ from the G3PDH reaction with increasing time and decreasing pH, and the opposite profile for the ATPase reaction.

Table 3 presents the ~H^+^_te_ of each reaction of cytosolic energy catabolism for the exercise and cellular pH conditions modelled in this study. The two largest ~H^+^ releasing reactions are the G3PDH and ATPase reactions respectively. The main metabolic ~H^+^ consumers are the LDH, PK, and CK reactions respectively.

Total H^+^ release from energy catabolism equaled −187.86 mmol L^−1^, metabolic H^+^ buffering was 130.58 mmol L^−1^, and given the single unitary change in cellular pH for this model (pH 7.017–6.018) total buffering capacity (total H^+^ release to pH change ratio) equated to 187.86 Slykes. The total ~H^+^ release to lactate accumulation ratio was 4.27 (187.86/44). Use of the now outdated and incorrect stoichiometric lactate derivation of H^+^ release would have revealed an erroneous buffer capacity of 44 Slykes (ΔLa to ΔpH ratio); an error factor equal to the H^+^ release to [La^−^] ratio.

Each of NAD^+^ balance and ADP balance revealed large changes of −12.63 and 24.63 mmol L^−1^ respectively. For NAD^+^ balance, this means that there was a decrease in NAD^+^ of this amount by the end of the exercise bout, which then means that the relative redox potential would have had a 25.26‐fold decrease. For ADP balance, this is a remarkably large ADP release which clearly does not result in an appreciable ADP accumulation, and such a result discrepancy is explained in the Discussion.

## 4 DISCUSSION

This research used prior published data of muscle metabolite accumulation in combination with prior documentation of the pH dependent multiple competitive cation ~H^+^_e_ for all chemical reactions of non‐mitochondrial energy catabolism to model cytosolic M_f_ during 3 min of intense exercise to contractile failure. Data for M_f_ and ~H^+^_e_ was used to calculate the H^+^_te_ for all pertinent reactions. The results revealed an appreciably large ~H^+^_te_ for each of glycolysis and ATP hydrolysis, and a fourfold larger total ~H^+^ release to lactate ratio than previously assumed from the lactic acidosis construct. The most ~H^+^ releasing reaction of glycolysis was the G3PDH reaction, which involves a more complex understanding of cellular inorganic phosphate metabolism than currently acknowledged. It will be argued that this research provides the most accurate and thorough quantification of cellular ~H^+^_e_ to date and the implications of these results to the biochemistry of cellular energy catabolism and metabolic acidosis will be discussed in detail.

The Discussion content to follow will be structured by metabolic energy system, with each consisting of M_f_ and then ~H^+^_e_. Discussion will then be directed to the implications of these results to the biochemistry of metabolic acidosis, past theories that have been proposed to cause metabolic acidosis, and finally to the limitations of this study.

### 4.1 Phosphagen system

There is extensive research on muscle creatine phosphate (CrP) degradation during exercise of different intensities, as well as CrP regeneration during the exercise recovery interval (Cannon et al., 2014; Kemp, 1997, 2001; Kemp et al., 2006, 2007; Layec et al., 2009, 2013, 2016; Moll et al., 2017; Slade et al., 2006; Sleigh et al., 2016; Wackerhage et al., 1996; Whipp et al., 1999). While the profile of CrP degradation would be dependent on the time and intensity profile of the exercise condition, during and in recovery from intense exercise to moderate intensity steady state or volitional exhaustion the decrease and recovery replenishment of CrP follow mono‐exponential profiles (Layec et al., 2013; Moll et al., 2017; Slade et al., 2006; Whipp et al., 1999). Data from ^31^P MRS documents larger concentrations of resting muscle CrP (30–40 mmol L^−1^ muscle water) than research based on muscle biopsy (15–20 mmol L^−1^), and this is understandable given the invasiveness and inherent delays in tissue sample excision and freezing for the biopsy procedure (Kemp et al., 2007). Consequently, the rest concentration and exercise‐induced depletion of muscle CrP followed the data profiles of these prior studies.

The phosphagen system is clearly net ~H^+^ consuming, and this capacity is mostly driven by the CK reaction (Table 1) which accounted for 80.7% of the ~H^+^ consumption of this system, with negligible ~H^+^ release from the AK reaction. Prior research has documented a slight alkalization of contracting skeletal muscle during the initial seconds of intense muscular contraction, and such changes have been ascribed to the CK reaction (Cannon et al., 2014; Haseler et al., 1998; McCann et al., 1995; Moll et al., 2017; Slade et al., 2006; Yoshida & Watari, 1992). As such, there is a documented history within research of muscle metabolism during intense exercise to infer direct involvement of chemical reactions to altering cellular pH. This issue will be raised again in the sub‐topic pertaining to past theories of cellular acid‐base balance.

### 4.2 Glycogenolysis, glycolysis, and lactate production

Since the Nobel Prize research of Meyerhoff on the glycolytic pathway (Raju, 1998; Shampo & Kyle, 1999), research of cellular glycolysis has occurred in a wide range of different tissues and lifeforms. For skeletal muscle metabolism during exercise, considerable research based on muscle biopsy methodology has been influential in enhancing our understanding of human muscle metabolism during intense exercise. As previously explained, key studies from this work were used to formulate the methods of this research. Details of the methods used in these studies will be provided in latter sections of the Discussion pertinent to specific results of this research.

The data of Table 3 reveal progressively lower values for M_f_ for the reactions of glycogenolysis and glycolysis as you sequentially proceed toward lactate production. This is logical based on the gradual product accumulation for each reaction over time during exercise leading to contractile failure and volitional fatigue. While lactate is the final product that accumulates from these pathways in muscle during repeated intense contractions, the reality of metabolic flux is that preceding reactions of a pathway all occur to allow this final product accumulation. This is not only true for lactate production and accumulation, but also relevant for ATP turnover when the [ATP] is stable or there is a gradual decrease in [ATP]. The same is true for Pi metabolism in contracting muscle, and these metabolites will be discussed in their respective sections.

Glycogenolysis is net ~H^+^ releasing, and as presented in Results, this is largely due to the role of Pi as a substrate in the phosphorylase reaction which releases ~H^+^ during its addition to the cleaved glucose residue from glycogen to form G1P. Furthermore, as shown in Figure 4, as pH decreases from 7.0 toward 6.0, the ~H^+^_e_ for the phosphorylase reaction becomes increasingly more negative. Such ~H^+^ release is not more prominent because it is constrained by the relatively large ~H^+^ association of G1P as pH falls (Robergs, 2017). In addition, the ~H^+^_te_ is further constrained by the lower M_f_ compared to the 3‐carbon metabolite reactions of phase‐II of glycolysis.

The results of Table 3 reveal the reaction involvement of glycolysis and how each reaction contributes to ~H^+^_te_. In addition, Table 3 documents the ~H^+^_e_ of specific reactions of glycolysis, and the most important reactions to ~H^+^ release were highlighted in Results.

### 4.3 Cellular ATP turnover

The capacity of ATP hydrolysis within living tissue is not measured by the decrease in the cellular [ATP]. Cell work is being performed continuously, and for contracting skeletal muscle, this work is appreciable and induces a continual ATP_to_ during stable cellular [ATP] to ensure sustained cell work and cell life. The different energy systems of a cell function to stabilize cellular [ATP] as close as possible to the resting value, which for human skeletal muscle approximates 8.2–10.0 mmol L^−1^ muscle water (~7 mmol kg^−1^ wet wt). As such, the resting cellular [ATP] is not an energy store, as it is essential to stabilize the concentrations of all the phosphorylated adenylate molecules (ATP, ADP, AMP) to ensure sufficient free energy release from ATP hydrolysis. This has been repeatedly verified by the relatively meagre reductions (<30%) in cellular [ATP] despite extreme conditions of voluntary and involuntary contraction‐induced ATP demand (Spriet et al., 1987a). Large perturbations of the concentrations of these adenylate molecules would compromise free energy release and add to other mechanisms of contractile failure.

**FIGURE 5:**
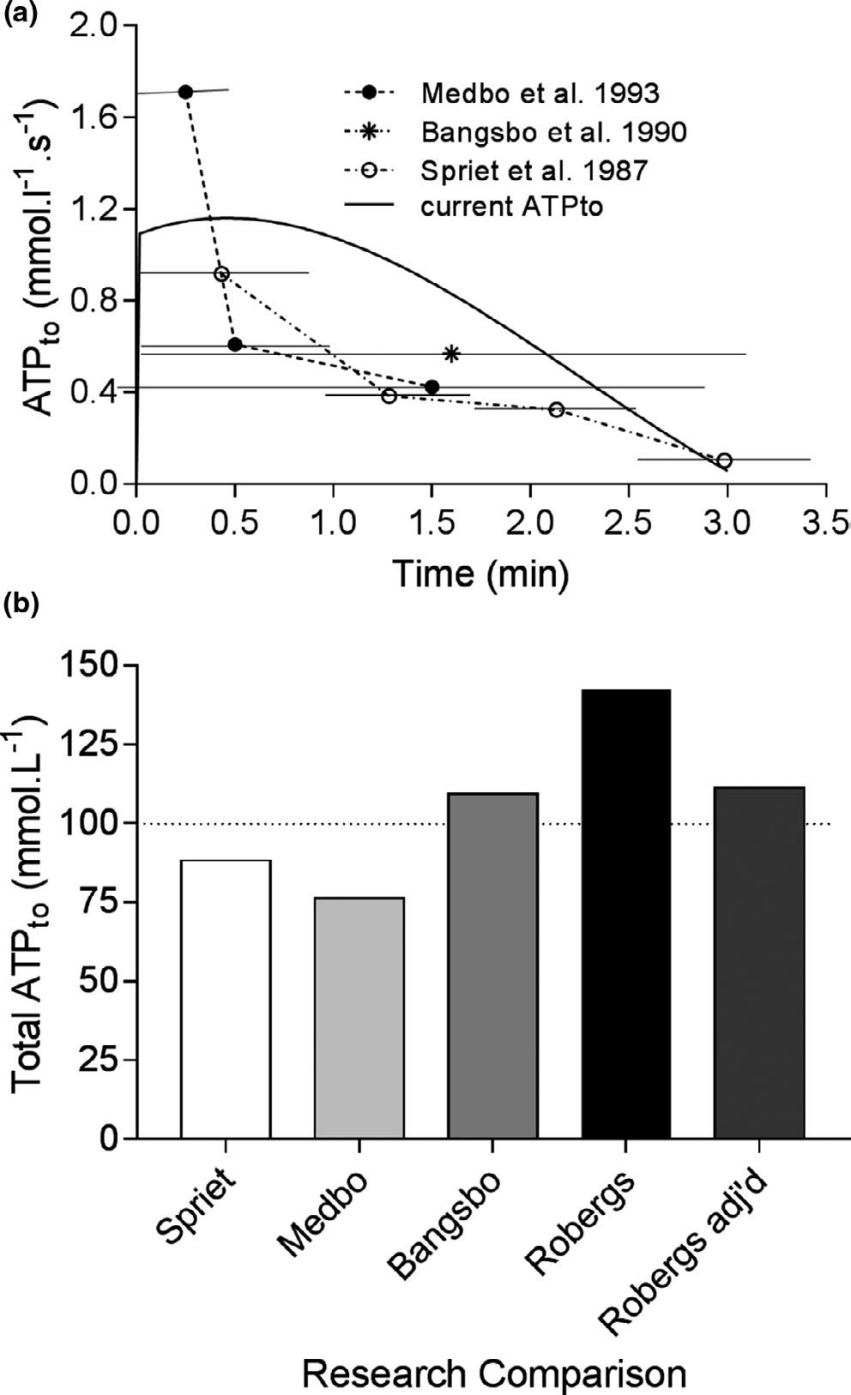
(a) Comparisons in data for ATP_to_ from the current study to that of Spriet et al. (1987a), Medbo and Tabata (1993), and Bangsbo et al. (1990). Horizontal lines represent the time duration for the data sampling and calculations. Symbols are placed at the median time value for this sampling window. (b) Due to the differences in methodology between studies, results are best compared for the variable of total ATP_to_. When the current data are adjusted (adj'd) for the ATP involvement in the PFK reaction the ATP_to_ becomes more similar across the four studies. “Robergs” within the *x*‐axis of Figure 5b refers to the current study. See Discussion for further explanations

There is remarkable external validity verification of the exercise model and methodology used in this research by the computation of ATP_to_ and the similarity these values have to prior research based on muscle biopsy (Bangsbo et al., 1990; Medbo & Tabata, 1993; Spriet et al., 1987a). Such data are presented in Figure 5a,b. For example, Spriet et al. (1987a) performed the most invasive and arduous protocol of intense exercise to contractile failure by exposing humans to artificial stimulation of the quadriceps femoris muscle. Muscle biopsies were acquired after 16, 32, 48, and 64 contractions (25.6, 51.2, 76.8, 102.4 s for summed contraction duration respectively). While the peak (averaged across the contraction time of the initial 16 contractions) ATP_to_ from Spriet et al. (1987a) was higher at 1.84 mmol L^−1^ s^−1^ than reported here at 1.16 mmol L^−1^ s^−1^, this is understandable given the use of contraction time rather than total time for the ATP_to_ calculations. To provide a valid comparison to volitional exercise, total time needs to be the reference integral condition. When these data are adjusted for total time the rate values are halved to a peak ATP_to_ of 0.92 mmol L^−1^ s^−1^ (Figure 5a). Furthermore, each of Spriet et al. (1987a), Bangsbo et al. (1990), Gray et al. (2011), and Medbo et al. (1993) used a crude estimation of ATP_to_ from similar, though different equations; ATP_to_ = ((1.5 × Δ[La^−^]) + Δ[CrP] + (2 × Δ[ATP]) − Δ[ADP])/time (s) (Spriet et al., 1987a), ATP_to_ = (1.5 × Δ[La^−^] + Δ[CrP] + Δ[ATP])/time (s) (Bangsbo et al., 1990; Medbo & Tabata, 1993), ATP_to_ = (1.5 × Δ[La^−^] + Δ[CrP] + (2 × Δ[ATP]))/time (s) (Gray et al., 2011).

The slight discrepancies between these equations are problematic due to subsequent differences in validity. However, the more important concern pertains to the lactate constant of 1.5 used in these equations, as this is an adjustment of the gross 4 ATP yield from the production of 2 La^−^ based on the ATP cost of the PFK reaction. The adjustment results in a net 3 ATP yield for each glucose conversion to 2 lactate, or the assumed 1.5 ATP per lactate. Yet to correct total cellular ATP production for the PFK reaction is an error that causes an under‐estimation of the ATP_to_. The ATP cost of phase‐I of glycolysis (during intense exercise this cost only involves the PFK reaction as the predominant source of G6P is from glycogenolysis and therefore the hexokinase reaction can be ignored) is a form of chemical work, and ATP_to_ should be based on the total amount of ATP regeneration from energy catabolism. The value for ATP_to_ from this study exceeded all prior integrated totals from the pertinent studies presented in Figure 5, but when adjusted down for the PFK reaction (which once again should not occur), was almost identical to that of Bangsbo et al. (1990), and very close to the data from Spriet et al. (1987a) and Medbo et al. (1993) who based their calculations on this adjustment error. Once again, the similarity of such comparisons of ATP_to_ across multiple prior research studies and researchers provides remarkable external validation of the methods and data results of this research.

Researchers who have focused on ATP hydrolysis as a potential major contributor to net cellular ~H^+^_e_ during intense muscle contractions (Dennis et al., 1991; Gevers, 1997; Gevers, 1979; Hochachka & Mommsen, 1983; Robergs et al., 2004; Zilva, 1978) have been criticized based on either the known limited change in muscle [ATP] during intense exercise or the dramatic reduction in the ~H^+^ release by ATP hydrolysis as pH decreases (Kemp, 2005; Kemp et al., 2006). For this modelled research, ATP hydrolysis accounted for the release of 56.39 mmol H^+^.L^−1^ muscle water (Table 3), which was shown to predominantly occur early in to the exercise bout and remain ~H^+^ releasing through the initial 2 min of exercise. It is noteworthy that ATP hydrolysis was the second largest contributor to ~H^+^_te_ to the glyceraldehyde‐3‐phosphate dehydrogenase reaction (61.51 mmol H^+^.L^−1^ muscle water). Clearly, ATP hydrolysis has a meaningful capacity of ~H^+^ release in transition from neutral and acidic cellular conditions and should not be overlooked as a potential contributor to the development of metabolic acidosis.

### 4.4 Cellular Pi turnover

Increased intracellular concentrations of inorganic phosphate ([Pi]; [HPO_3_^−2^]) occur from an imbalance in ATP turnover (excess) and mitochondrial ATP regeneration (limited), where the latter is an important consumer of Pi as a substrate in oxidative phosphorylation. During cytosolic energy catabolism, Pi is also a substrate for the phosphorylase and G3PDH reactions. The data of Table 3 reveal that for the exercise condition of this research, gross Pi release from ATP hydrolysis computed to 111.35 mmol L^−1^ (the PFK corrected value is used here as the ATP used in this reaction is not available for the ATPase reaction) and that Pi consumption during glycogenolysis and glycolysis equated to 91.61 mmol L^−1^, revealing a net Pi release of 19.74 mmol L^−1^.

The relatively small Pi accumulation calculated for this modelling represents just 63% of the decrease in CrP, and only 18% of the PFK adjusted total ATP_to_. However, before comparisons to past research, it is important to clarify the differences in absolute concentrations of phosphate metabolites obtained from muscle biopsy research versus research from 31‐phosphorous magnetic resonance (^31^P MRS) methodologies.

For research involving ^31^P MRS, it is important to acknowledge the influence of magnetic field strength. Early research (pre‐2000) of ^31^P MRS was confined to magnetic field strengths <2 Tesla, whereas in recent years it is now common to see use of large bore magnets between 3–10 Tesla. For a given tissue sampling volume, the larger the magnetic field strength the greater the resolution of peaks for different phosphate metabolites, as well as the increased signal intensity for each metabolite and more rapid nuclear relaxation time, thereby allowing higher signal to noise, higher acquisition frequencies, reduced averaging of spectra and thereby improved temporal resolution. Whereas early studies required up to 1–2 min of signal averaging at anywhere from 2 to 5 s acquisition intervals, current high field strength studies now produce quality data with approximately 6–15 s of spectra averaging at 1–2 s acquisition intervals (Kemp et al., 2007; Layec et al., 2009, 2013, 2016). Increasing the sample volume further improves all these signal quality features of ^31^P MRS data acquisition. The relevance of this information is that data should be of greater quality in studies using higher field strength magnets, with further improvement at all magnet field strengths when using large sample volumes, and such methodological conditions will be referred to when discussing results from pertinent ^31^P MRS studies.

Research from ^31^P MRS cannot directly quantify metabolites. To do so, researchers assume that resting muscle [ATP] is ~8–10 mmol L^−1^, that total creatine functions as a closed pool and equals 42.5 mmol kg^−1^ wet wt. (Kemp et al., 2007; Taylor et al., 1986), and that muscle [ADP] can be calculated from the creatine kinase equilibrium (1.66 × 109 M L^−1^ at ionic strength = 0.25, free [Mg^2+^] = 1 mmol L^−1^, and muscle temperature = 38°C). Added to these assumptions is the difficulty in resolving the pure Pi peak for intense exercise conditions, and this is especially true for lower magnetic field strength magnets, as the increasing accumulation of the hexose and triose phosphate intermediates of glycolysis have resonance frequencies close to that of Pi and as such appear as a broad shoulder off the Pi peak, though this resolution is improved in higher magnetic field strength magnets.

When reading past research from ^31^P MRS of muscle metabolism during intense exercise, there are large discrepancies in the extent of muscle Pi accumulation. For example, Taylor et al. (1986) reported Pi accumulation after 4 min of intense bulb squeezing (finger flexion focused on the small flexor digitorum superficialis) in a 1.9 Tesla magnet to be 42 mmol L^−1^ muscle water, quantified by an assumed internal standard of the β‐ATP peak of 8.2 mmol L^−1^ muscle water. For the same conditions, [CrP] decreased from 38.2 to 6.4 mmol L^−1^ muscle water, revealing a Pi accumulation relative to CrP decrease (Δ[Pi]/Δ[CrP]) of 119.5%. More recent research from ^31^P MRS has revealed peak Pi accumulation during intense exercise that is considerably lower than that of Taylor et al. (1986). For example, Layec et al. (2009) reported a relative Pi (to CrP) accumulation of 61% for intense exercise at 35% of the MVC for two‐legged knee extension, with ^31^P MRS signal acquisitions using a large 14 cm surface coil place over the right rectus femoris quadriceps muscle within a 1.5 Tesla magnet. Free induction decays (FIDs) were acquired every 2 s (each contraction cycle gated to end recovery), and each spectra was an average of 4 FIDs, resulting in an 8 s time average. Similarly, Layec et al. (2016) reported a relative Pi (to CrP) accumulation of 67% for intense exercise involving isometric maximal voluntary contraction (MVC) knee extension sustained for 24 s within a 2.9 Tesla magnet using a 125 mm surface coil placed under the quadriceps (rectus femoris) muscle with the subject lying prone. FIDs were acquired every 2 s, with each spectra an average of 3 FIDs across a time window of 6 s. The similarity of the results of Layec et al. (2009, 2016) to this study are impressive and reveal concern over the methods used to remove the signal from phosphorylated metabolites from the Pi quantification in the Taylor et al. (1986) study. A quantitatively different Pi accumulation to CrP decrease is to be expected given that the two metabolites are not directly biochemically connected, as previously explained. In addition, such differences are further evidence of the invalid nature of using the Lohman reaction to fictitiously couple the CK and ATPase reactions to simplify interpretations of cellular energy catabolism during intense or short‐term repeated muscle contractions (Broxterman et al., 2017; Kemp, 1997, 2001; Kemp et al., 2006, 2007).

The metabolic role of Pi becomes even more interesting when you compute the pH dependent ~H^+^_e_ for the phosphorylase and G3PDH reactions. As pH falls, the Pi released from ATP hydrolysis is now more fractionally protonated (from HPO_4_^−2^ to H_2_PO_4_^−1^), and this can be seen from the G3PDH reaction H^+^_e_ curve of Figure 4. In other words, a fall in cellular pH toward 6.0 increases the fraction of H_2_PO_4_^−1^ from 0.3599 at pH 7.0 to 0.849 at pH 6.0. This ~H^+^ association of Pi increases the ~H^+^ release for each of the reactions where Pi is a substrate (phosphorylase and G3PDH). For sustained intense exercise to contractile failure and volitional exhaustion, as modeled in this research, the anapleurotic nature of Pi in cellular energy catabolism complicates how to interpret the buffering nature of Pi, as the integral of the ~H^+^_e_ of the phosphorylase and G3PDH can cause a net ~H^+^ release that exceeds the perceived ~H^+^ buffering by Pi accumulation.

### 4.5 Cytosolic redox and [ADP]

Glycolysis produces NADH, while lactate production oxidizes NADH and reduces pyruvate to lactate, regenerating cytosolic NAD^+^. How do these competing rates of NADH and NAD^+^ appearance and disappearance influence net cytosolic NAD^+^/NADH proportions (redox) once the specific substrate flux through these reactions are integrated over time? Based on the observed increase in substrate flux for preceding reactions to the LDH reaction as you backtrack along phase II of glycolysis, more NADH is produced from the G3PDH reaction than NAD^+^ regenerated from the LDH reaction. As such, it is simple biochemical logic for the NAD^+^ to NADH redox ratio to gradually fall and remain low as lactate production increases. This deficiency in lactate coupled NAD^+^ regeneration integrates to a 24‐fold relative change at the end of the exercise condition. This relative decrease in cytosolic NAD^+^ is likely to be a significant contributor to a gradual decrease in substrate flux through glycolysis during intense exercise, which in‐turn would gradually compromise ATP_to_ necessitating the gradual decrease in muscle [ATP] with simultaneous increases in [Pi], [ADP], and contractile failure.

The calculated increase in cytosolic ADP release for the exercise condition of interest was 24.7 mmol L^−1^. This is remarkably large and no research of muscle metabolism during intense exercise has calculated intramuscular ADP accumulation anywhere near close to this extent. As explained for muscle ATP metabolism, a cell would not tolerate such increases in [ADP] based purely on drastic alterations of the free energy transfer of ATP hydrolysis, or indeed any reaction where ADP is a product. Clearly, the cellular handling of ADP is complex, and it is assumed that cellular mitochondria must act as a sink for ADP during intense muscle contractions. To some extent, this is logical as there would then be a readily available supply of ADP substrate, and presumably also Pi and H^+^ for oxidative phosphorylation to respond to the improved oxygenation from increased blood flow and perfusion during the recovery from intense muscle contractions. Such a rapid response ATP supply is also important for the rapid regeneration of CrP by the CK reaction in the immediate post‐exercise recovery period. Perhaps this is further evidence for the importance of the creatine kinase shuttle in connecting phosphate transfer between the mitochondria and the cytosol (Bessman & Greiger, 1981). In this instance, after intense exercise, the rapid increase in mitochondrial ATP regeneration combined with the relatively higher cytosolic ADP both combine to increase cytosolic [CrP]. This in turn allows for the transfer of Pi from CrP to cytosolic ADP to eventually provide ATP throughout the cell. Of course, such Pi transfer occurs during all variances of cellular ATP demand. To some extent, this function may have been indirectly documented in the research of high intensity exercise priming of the metabolic pathways causing improved VO_2_ kinetics during subsequent transitions from rest to moderate intensity steady state exercise (Burnley et al., 2011; Maturana et al., 2018).

### 4.6 Implications to past theories of the cause of metabolic acidosis

Given that cells do not produce lactic acid, the only remaining theory of the cause of metabolic acidosis is Peter Stewart's physico‐chemical theory (Corey, 2003; Morgan, 2009; Stewart, 1978, 1983). Stewart first introduced this theory in 1978 (Stewart, 1978) with further scientific clarification in 1983 (Stewart, 1983). The theory is based on the fundamental principle that in a solution all charged components (ions, metabolites, etc.) must sum to equal zero charge. In summary, Stewart's approach proposed that the ionic shifts that occur in contracting muscle, or in systemic biological fluids caused by cellular cation release, also cause alterations in charge distribution. Stewart argued that all solutions conserve charge neutrality, thereby revealing that when there is a gain in net negative charge there is a force that will cause ~H^+^ release to ionize water to form H_3_O^+^, which by definition will then be reflected as a decrease in pH and contribute to restoring charge neutrality. Presumably, other cations would also be involved in this electro‐chemical response, so the relatively minor H^+^ adjustment in [H_3_O^+^] is not a concern. Nevertheless, Stewart did not provide any experimental biological evidence for his underlying theory of electrochemical neutrality, he did not explain where these H^+^ come from, nor did he provide evidence for any of his assumptions for this model to independently (no additional source or cause of the H^+^ change) regulate the pH of biological fluids.

This latter issue is crucial to the biological impact of this research. Stewart was adamant in the [H_3_O^+^] of biological solutions being a dependent variable. In other words, there was no cellular or systemic source of H^+^ separate to this electro‐chemical correction (see Introduction) (Stewart, 1983). Thus, according to Stewart, chemical reactions might release ~H^+^, but these H^+^ do not influence the pH of biological solutions, which is to say that despite chemical ~H^+^_e_ during chemical reactions, for some reasons these H^+^ are different to those coming from strong acids or from whatever the source is that functions within the physico‐chemical construct. At that time, there was no evidence for the capacity of the ~H^+^_e_ from the chemical reactions of cellular energy metabolism, and presumably most scientists agreed that if H^+^ release did occur it could only be from lactic acid. Yet, for the first time, the results of this research clearly show that such ~H^+^_e_ is not minor, but more correctly labeled as ‘unexpectedly large’; though numerous scientists have been proposing this since the late 1970's (Dennis et al., 1991; Gevers, 1977; Gevers, 1979; Hochachka & Mommsen, 1933; Zilva, 1978) but devoid of the computational complexity of this research.

(11) $$K_{w}=\left[H^{+}\right]\left[{\text{OH}}^{‐}\right],$$

(12) $$K_{d}\text{HA}=\left[H^{+}\right]\left[A^{‐}\right],$$

(13) $$\left[A_{\text{tot}}\right]=\left[\text{HA}\right]+\left[A^{‐}\right],$$

(14) $$K_{c}{\text{PCO}}_{2}=\left[H^{+}\right]\left[{\text{HCO}}_{3}^{‐}\right],$$

(15) $$K_{3}\left[{\text{HCO}}_{3}^{‐}\right]=\left[H^{+}\right]\left[{\text{CO}}_{3}^{2‐}\right],$$

(16) $$0=\left[\text{SID}\right]+\left[H^{+}\right]‐\left[{\text{HCO}}_{3}^{‐}\right]‐\left[A^{‐}\right]‐\left[{\text{CO}}_{3}^{2‐}\right]‐\left[{\text{OH}}^{‐}\right].$$

Finally, there are also concerns over the mathematical principles of the Stewart SID approach in computing pH change. Equations (11)–(16) present the main equations used by Stewart to estimate pH (Stewart, 1978, 1983). There is some irony that K_d_'s are used within many equations yet the K_d_'s of cellular or extracellular metabolites are ignored. Indeed, only one acid‐base entity is factored into this computational model, and this represents the net sum of all weak acid behaving metabolites, though Stewart interpreted this to be equivalent to the total protein concentration of a solution (Equation 13). Based on the results of this investigation, this is a clear error and presents a computational component that is essentially impossible to measure. Then there is the component of the sum of all negatively charged weak acid bases, which Stewart proposed was an entity to be estimated and assumed simply to be the [La^−^]. This is a major flaw of this computational model because as we now know from these data, and that from Robergs (2017), the negative charge resulting from energy catabolism is complicated by pH and would be equivalent to the net change in all metabolite accumulation profiles adjusted by each of the metabolite pH and log K_d_ dependent values. Though lactate is the largest single accumulating metabolite, the net summation of all other metabolites from this research at the end of the modeled exercise conditions was estimated to be 83.1 mmol L^−1^; more than double the M_a_ for lactate. The charge characteristic of this entity was not computed in this research but can be done from this methodology and is clearly a needed topic for an additional manuscript. These concerns reveal the difficulty of applying the Stewart method to cellular acid‐base balance and may also partially explain the large error documented for the Stewart method of the pH estimation of blood (Kowalchuk & Scheuermann, 1994).

Peter Stewart has to be acknowledged for his attempt at explaining body fluid pH based on physico‐chemical principles, but is it clear that despite some clinical acceptance of this approach (Corey, 2003; Morgan, 2009), the model is deficient in accounting for all features of cellular and systemic body fluids that can influence ~H^+^_e_ and therefore the pH of body fluids. A new model is needed to improve the construct validity of understanding metabolic acidosis and perhaps the broader acid‐base regulation of biological systems. This approach would need to account for cellular determinants to intracellular pH and the cell to extracellular transport of H^+^. In this context, there needs to be experimental verification or refutation of many of the assumptions of the Stewart approach, and based on this evidence, a new computation model can be developed.

### 4.7 Limitations of this study

The duration of intense exercise was chosen to be 3 min due to repeated studies documenting the validity of this duration to the maximal accumulated oxygen deficit and muscle anaerobic capacity (Bangsbo et al., 1990; Medbo & Tabata, 1993; Spriet et al., 1987a). Such a relatively long duration could be questioned, but the duration of the exercise condition used in this modelling is of minimal concern. The metabolic flux calculations are based on prior research of muscle metabolite accumulation, and especially the well‐researched entity of muscle lactate accumulation. Shortening the exercise duration while retaining the change in cellular pH and metabolite accumulation would still yield similar data for each of ΔM, M_tf_, and H^+^_te_, and such preliminary evidence across a shortened exercise duration to 1.5 min has already been published (Robergs, 2019). A decreased exercise duration increased the peak rates of flux for metabolites (M_f_) and H^+^ (~H^+^_e_) in inverse proportion to the exercise duration, with minimal influence on the integral of these measures. The minimal changes that did occur were because of how changes in the temporal profile of M_f_ for each metabolite and cellular pH differed to their relative profiles of this study.

Added limitations pertain to the confinement of the calculations to reactions of the phosphagen and glycolytic catabolic energy systems. Mitochondrial respiration is a known consumer of H^+^ through the final reaction of the electron transport chain, as well as in the generation of the mitochondrial intermembranous space:matrix H^+^ gradient (Krebs et al., 1975). There are also other ancillary reactions that occur in the cytosol. However, during intense exercise the prior research used to establish metabolite concentrations explained in Methods and support result interpretation in the Discussion are clear in focusing on the impact of the phosphagen and glycolytic catabolic energy systems as the primary determinants to cellular metabolic conditions impacting ATP turnover and H+ balance. That said, the transition from pH neutral to acidic conditions within skeletal muscle is proposed to be a key determinant to endurance exercise performance and future research and modeling needs to eventually tackle the complex issue of how the cytosol and mitochondria combine to influence cellular H^+^ balance.

The function of the monocarboxylate transport protein would facilitate lactate efflux from muscle to the interstitial space, even during anoxic ischemic conditions. As such, there is logic to viewing the lactate accumulation and related ~H^+^_e_ data for the LDH reaction and the glycolytic pathway to underestimate true values. As it remains unknown what this extent of added lactate sink is, or how its fluid volume changes with different intensities and durations of exercise, no attempt was made to account for this space in computations of reaction or pathway metabolite flux.

Finally, it is important to realize that the results of this research do not provide any answers to the cause of cellular or blood acidosis. The data reveal that the ~H^+^_e_ of chemical reactions is larger than previously understood. Whether such ~H^+^ involvement can directly alter the pH of body fluids remains to be verified.

## 5 CONCLUSIONS

The lactic acidosis construct has been an accepted component within acid‐base chemistry and physiology, and related topics, for more than 100 years. Such acceptance had prevented the need to research alternative causes of ~H^+^_e_ from other chemical reactions. Based on applications of computational chemistry, this research has quantified ~H^+^_e_ during the reactions of skeletal muscle non‐mitochondrial energy catabolism. Using muscle metabolite accumulation from prior research, computation of reaction specific ~H^+^_e_ at given cellular pH revealed an appreciable total ~H^+^ release that equated to −187.9 mmol L^−1^. ATP hydrolysis involved in cellular ATP_to_ accounted for −47.5 mmol L^−1^ (25%) of this ~H^+^ release. New insights were also provided on the capacity of ~H^+^ release and consumption for specific reactions of the phosphagen, glycogenolytic, and glycolytic energy systems. We now know that the ~H^+^_e_ from chemical reactions during times of high metabolic demand are remarkably large, and that despite considerable ~H^+^ consumption, cellular energy catabolism during intense exercise exceeding mitochondrial capacities is net ~H^+^ releasing, with the exact numerics of the release being dependent on cellular pH and the extent of mitochondrial deficiency.

The data may have important implications to understanding the cause of metabolic acidosis coincident with increased cellular metabolic demand. Nevertheless, considerably more research is required to establish how the ~H^+^_e_ of chemical reactions may or may not be involved in the determination of the cellular and systemic acid‐base balance of biological solutions.

## CONFLICT OF INTERESTS

The author has no competing interests or conflicts of interest in the completion of this research and publication of the manuscript.
